# Turning Pineapple Crown Waste Into Value: Formulation and Analysis of an Instant Powder Beverage

**DOI:** 10.1002/fsn3.71220

**Published:** 2025-11-16

**Authors:** T. B. N. Brito, A. B. Koifman, E. M. Antonio, M. G. O. Carvalho, F. S. N. Cardoso, J. L. Bicas, A. E. C. Fai, M. S. L. Ferreira

**Affiliations:** ^1^ Laboratory of Bioactives (LABBIO), Food and Nutrition Graduate Program (PPGAN) Federal University of State of Rio de Janeiro (UNIRIO) Rio de Janeiro Brazil; ^2^ Department of Food Science and Nutrition, School of Food Engineering University of Campinas (UNICAMP) Campinas Brazil; ^3^ Laboratory of Multidisciplinary Practices for Sustainability (LAMPS), Institute of Nutrition State University of Rio de Janeiro (UERJ) Rio de Janeiro Brazil

**Keywords:** antioxidants, freeze‐drying, microencapsulation, morphology, spray‐drying, waste

## Abstract

Pineapple processing generates significant waste, with the crowns accounting for 28%. The crowns are rich in nutrients and phytochemicals and offer potential for high value‐added bioactive products such as herbal infusion. Therefore, this work aims to develop and characterize a microencapsulated bioactive beverage powder from pineapple crown infusion. The crowns were dried, ground, and added to water (90°C; 10 min) to obtain the infusion. The microcapsules were obtained by adding maltodextrin to the infusion and drying by freeze‐drying (FD) or spray‐drying (SD). Physicochemical parameters, morphology, encapsulation efficiency (EE), total reducing capacity (TRC), antioxidant capacity (AC), and phenolic compound content (HPLC‐DAD) were determined. The microcapsules had an average size of 12–166 μm and similar pH (~4.3), Aw (< 0.40) and moisture content (~3.5%). The microcapsules were highly soluble (99.98%) with wettability 1 (FD) at 191 s (SD). The sorption isotherms were of the J type. FTIR revealed similar bands between the two samples, with characteristic bonds of hydroxyl groups, carboxylic acids, alcohols, and aromatic rings. SD showed higher TRC but similar AC to the FD samples. The EE for both drying techniques was ~98%. Core extracts showed greater antioxidant capacity than surface extracts. Three hydroxycinnamic acids (p‐coumaric, *trans*‐ferulic, and caffeic acid) were identified. The reconstituted beverage (SD) showed a higher content of phenolic acids than the infusion PCF and FD microcapsules, with p‐coumaric acid being the most abundant. The pineapple crown soluble powder drink has promising industrial characteristics and proposes using a part of the fruit discarded in conventional production practices.

## Graphical abstract

The pineapple crown, a by‐product representing 28% of processing residues, is rich in nutrients and phytochemicals with potential for bioactive applications. This study developed and characterized a microencapsulated beverage powder from crown infusion using freeze‐drying (FD) and spray‐drying (SD). Both techniques produced soluble powders (~99.98%) with low moisture, stable physicochemical properties, and high encapsulation efficiency (~98%). SD samples showed higher phenolic acids, especially p‐coumaric. Results highlight the industrial potential of pineapple crown powders as value‐added functional ingredients.

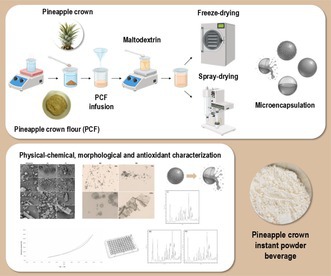

## 1 Introduction

Pineapple ( *Ananas comosus* ), a symbol of tropical and subtropical regions, is widely appreciated worldwide in its natural and processed form. Grown in all states of the country (EMBRAPA 2022) and originating in South America, pineapple is one of the most consumed edible members of the Bromeliaceae family and grows predominantly in places with hot and dry climates. In 2022, global pineapple production was approximately 29.4 million tons. The fruit is the third most produced in Brazil, contributing approximately R$2.22 billion to the agricultural gross domestic product (GDP) (EMBRAPA 2022; FAOSTAT 2025). The pineapple's parts comprise a crown, core, pulp, and peel. The pulp, the usually edible part of the pineapple, corresponds to just over half of the weight of the fruit (51.2%). The crown (13.9%), the core (14.7%), and the peel (27.8%) are parts that are not usually consumed and are therefore considered pineapple waste. Of the total of this waste, the crown comprises 28.3% (Braga et al. 2015).

Because of the massive production of pineapple and related products, a significant amount of agro‐industrial waste is generated, including post‐harvest losses and waste from the food industry (Aili Hamzah et al. 2021). A survey carried out in Brazilian fruit and vegetable stores and supermarkets reported that more than 100 tons of fruits and vegetables were generated in 1 year, and that approximately 50% of this amount is waste that is discarded. Notably, pineapple has emerged as one of the primary sources of this waste, including peels, bagasse, and crowns (Brito et al. 2020b). In addition to the conventionally consumed edible portions, pineapple by‐products possess significant technological and bioactive potential, which is often lost when these parts are discarded. In addition to being rich in insoluble fibers, such as lignin, cellulose, and hemicellulose, the crown exhibits a rich composition of antioxidant compounds (Braga et al. 2015; Johny et al. 2023).

Dried pineapple crowns possess reduced moisture content and low water activity, providing enhanced stability, extended shelf life, and greater ease of handling and storage—key attributes for the development of beverages and other value‐added products. PCF demonstrated a rich profile of bioactive compounds such as *p*‐coumaric, ferulic, caffeic, 4‐hydroxybenzoic phenolic acids, and some lignans. Brito et al. (2021) found that these compounds are bound to their lignocellulosic matrix, and therefore, their potential may be related to the presence of antioxidant fibers. From the extraction of the essential oil of PCF by hydrodistillation, the volatile compound profile showed a rich composition of terpenic compounds such as α‐terpineol, α‐ionone, β‐farnesene, and linalool that are related to fruity aromas and bioactive properties such as antimicrobial and antioxidant activities.

In this context, recent studies have reported that inedible plant parts constitute valuable raw materials for the preparation of infusions, frequently exhibiting higher phytochemical concentrations than their edible counterparts (Acar et al. 2022). Infusions are among the most consumed beverages worldwide, ranking second to water. This is due to their attractive aromatic profile and their excellent health‐promoting effects, including anti‐inflammatory, cardiovascular, anticancer, antidiabetic, anti‐obesity, neuroprotective, and antioxidant activities. Infusions are prepared from flowers, fruits, seeds, leaves, roots, stems, and fruit peels heated in drinking water at over 90°C for a specific period according to the plant species (Finimundy et al. 2020; Studzińska‐Sroka et al. 2021). The benefits associated with the ingestion of infusions are generally related to the presence of bioactive compounds extracted from plants during this process. Different infusions contain a variety of phytochemicals, including lignans, sulfides, polyphenols, carotenoids, coumarins, saponins, phytosterols, curcumins, and phthalides (Wargovich et al. 2001).

However, the sensitivity of these bioactive compounds to external factors such as light, high temperatures, and storage conditions until the infusion process is a major disadvantage that hinders their practical use in the food industry and limits their applicability (Massounga Bora et al. 2018). Thus, encapsulation is one of the most promising techniques to maintain the bioactive properties of plant‐based extracts such as infusions, as it can protect bioactive compounds during processing, storage, and transportation. Protection occurs through the formation of a protective film by encapsulating agents on the compounds or substances to be stabilized. Capsules act by preventing active substances from degrading and losing their activities, protecting the most chemically unstable and easily oxidizable components and allowing an adequate release of these bioactive compounds, which can even increase their bioavailability (Nguyen et al. 2022; Ribeiro and Veloso 2021; Simsek and Süfer 2021). Therefore, this work aims to develop and characterize a microencapsulated bioactive beverage powder from pineapple crown infusion.

## 2 Material and Methods

### 2.1 Obtaining Pineapple Crown Flour and Infusion

Pineapple crowns ( *Ananas comosus* ) cultivar Pérola (*n* = 12) were obtained from commercial establishments in Rio de Janeiro and immediately defoliated and sanitized. They were cut and dried in a ventilated oven (Marconi, MA 035, Brazil) at 65°C for 12 h and dried again at 90°C for 1 h. They were then ground in a ball mill (SL‐38, Solab, Brazil) for 1 min, obtaining pineapple crown flour (PCF) (90.7% solid content). Finally, it was stored at 25°C in aluminum‐coated and airtight bags, protected from light (Brito et al. 2020a). PCF is characterized by reduced moisture content and low water activity, which confer enhanced physicochemical stability, extended shelf life, and improved ease of handling and storage. These attributes are particularly advantageous for ensuring the standardization and scalability required in the development of infusions and other value‐added products, reinforcing PCF as a reliable and sustainable raw material. For the infusion, 4 g of PCF was added to 100 mL of water initially at 90°C for 10 min; then the infusion (4%) was filtered through a paper filter and stored at −18°C for later analysis (Moraes et al. 2020).

### 2.2 Obtaining PCF Infusion Microcapsules

In the production of microcapsules, 5 g of maltodextrin (DE 17‐20, Inlab 5318, Brazil) was added to 100 mL of infusion and magnetically stirred (10 min; 1300 rpm) (Santiago‐Adame et al. 2015). The solutions were dried using lyophilization and spray drying techniques. For the lyophilization method, the solutions were initially frozen at −80°C for 24 h in an ultrafreezer (Indrel IULT 355D, Brazil) and subsequently dried in a benchtop lyophilizer (Terroni LD 3000‐D, Brazil) operating at 0 ± 0.3 Pa. The pressure change was monitored, and drying was completed after stability. For spray drying, the solutions were dried in a benchtop spray dryer (B‐290, BUCHI Corporation, New Castle, DE, USA), using an inlet temperature of 150°C, an outlet temperature of 100°C, and a pump at 20%. The feed and aspirator flow rates were set at 7 mL min^−1^ and 100%, respectively, in concurrent mode. The powders resulting from both processes were packaged in airtight aluminum bags and stored in the freezer at −20°C to be analyzed later.

### 2.3 Physicochemical Analyses of Microcapsules

#### 2.3.1 Moisture, Water Activity, and pH

The moisture content of the microcapsules was determined by the gravimetric method in triplicate at 105°C (AOAC, 925.10). Water activity (Aw) was determined using a portable water activity analyzer (Aqualab Pawkit, Meter, Brazil) in triplicate at 25°C. The pH of the samples was determined in triplicate using a benchtop pH meter (Gehaka PG2000, Brazil), with the samples dissolved in water at a concentration of 0.1%.

#### 2.3.2 Wettability and Solubility

Wettability was assessed according to the method described by Sun et al. (2020), with modifications. This determination estimates the time required for the complete submersion of a powder mass (50 mg) placed on the surface of a fixed volume of distilled water (20‐mL test tube) without stirring at 20°C in triplicate. Water solubility was determined according to Cortés‐Rojas et al. (2014) with adaptations. Therefore, 0.1 g of the sample was dissolved in 10 mL of distilled water under magnetic stirring (25°C; 10 min) and centrifuged at 6000× *g* at 25°C for 10 min (Heraeus Megafuge 16R, ThermoFisher Scientific, Germany). The supernatant (2 mL) was dried in an oven at 105°C for 5 h in a previously dried and weighed capsule. Solubility was expressed by the percentage of dry material solubilized and calculated according to the equation:

$$\mathrm{Sol}=\frac{\mathrm{ms}}{\mathrm{mi}}$$

where *ms* is the mass of dry solids in the supernatant, and *mi* is the mass of the initial sample.

#### 2.3.3 Sorption Isotherm

The sorption isotherms at 25°C were analyzed using an isotherm generator system (Aqualab Vapor Sorption Analysis, VSA, Decagon Devices, Meter, Brazil) using the “Dynamic Dewpoint Isotherm” (DDI) method. In this method, saturated or dry air is continuously inserted into the chamber, a dew point sensor measures water activity, and humidity is evaluated by weighing the sample during wetting. Approximately 500 mg of the microcapsule samples were added to the stainless steel capsules, and the analysis began on a “wet basis” with the initial humidity value of the sample (Brito et al. 2019). The isotherms were modeled according to the Guggenhein‐Anderson de Bôer (GAB) model using the equation:

$$M=\frac{m_{0}\times C\times K\times a_{w}}{\left(1-K\times a_{w}\right)\times \left(1-K\times a_{w}+C\times K\times a_{w}\right)}$$

where: *M*, equilibrium humidity (% dry basis); *a*_*w*_, water activity; *m*_0_, moisture in the molecular monolayer; *C* and *K*, parameters depending on the temperature and nature of the product.

### 2.4 Chemical Characterization by Fourier Transform Infrared Spectroscopy

Fourier transform infrared spectroscopy (FTIR) analysis was performed using an infrared spectrophotometer (IRPrestige‐21, Shimadzu, Japan) and the IRSolution acquisition software, version 1.60. Spectra were obtained in the range of 400–4000 cm^−1^ with a resolution of 4 (1/cm), 16 scans, Happ‐Genzel apodization, and a pure KBr tablet (spectroscopic grade) was used as a baseline (background). A ratio of 1:100 (sample:KBr) was used, with approximately 2 mg of sample: 200 mg KBr dispersed in an agate grail and pestle and transferred to a 13‐mm diameter stainless steel mold by a hydraulic press (model SSP‐10ª, Shimadzu, Japan) connected to a vacuum pump. A force of 80 kN was used for 10 min under a vacuum to form the tablet.

### 2.5 Characterization of the Morphological Properties of the Microcapsules

#### 2.5.1 Spatial Distribution of Microparticles by Fluorescence Microscopy

The distribution of the microparticles was analyzed by fluorescence microscopy (Axioscope 5, Carl Zeiss, Germany) in a bright field using 10× and 100× objectives. The images were captured with a camera (Axioscam 503 color, Zeiss, Germany), and the micrographs were processed using ImageJ software (version 1.53 n 7, National Institutes of Health, USA) (França et al. 2024).

#### 2.5.2 Morphological Analysis of Microparticles by Scanning Electron Microscopy

The micrographs were obtained by scanning electron microscopy (SEM) on a bench model (TM4000 Plus, Hitachi, Japan) using the TM4000 acquisition software. The samples were previously fixed on aluminum support and metallized with gold foil. The analyses were performed with an accelerating voltage of 10 kV and a beam current of 50,000 nA to obtain the micrographs. The magnitudes used varied between 50 and 2000 times.

#### 2.5.3 Particle Size and Diameter

The microcapsules' size distribution and mean diameter were analyzed according to Vardanega et al. (2019). The light scattering method used laser diffraction (Mastersizer 2000, Malvern Instruments Ltd., UK). The mean diameter was determined based on the mean diameter of a sphere of the same volume, the Brouckere diameter (D4.3), as described in Equation (1). Equation (2) below was used to calculate the polydispersity of the particles (Span). The samples were analyzed by the wet method, using ethanol (99.5%) as the dispersant. The obscuration values ranged from 5.55% to 5.85%, and the refractive index 1.36 was used for the dispersed phase (ethanol).

(1) $$D_{4,3}=\frac{\sum_{i=1}^{K}n_{i}\times D_{i}^{4}}{\sum_{i=1}^{k}D_{i}^{3}}$$

where: *D*_*i*_ is the mean particle diameter, and *n*_*i*_ is the number of particles.

(2) $$\text{span}=\frac{d_{90}-d_{10}}{d_{50}}$$

where *d*_90_, *d*_50_, and *d*_10_ correspond to an average diameter equal to 90%, 50%, and 10% of the accumulated volume, respectively.

### 2.6 Encapsulation Efficiency

Encapsulation efficiency was determined by extracting the capsules and determining the total reducing capacity (TRC, as presented in item 2.7.1). The calculation was performed according to the methodology described by Mujica‐Álvarez et al. (2020). For this purpose, the capsules were subjected to two distinct extraction processes. Firstly, the microcapsules were ruptured to allow quantification of encapsulated compounds. To this end, 0.375 g of microcapsules were mixed with 15 mL of 80% (*v*/*v*) methanol acidified with 0.1% HCl and sonicated in an ultrasonic bath (Ultrasonic Tank, Cristofóli, Brazil) for 30 min and centrifuged (4000× *g* for 5 min) (Heraeus Megafuge 16R, ThermoFisher Scientific, Germany). In the second step, the compounds present on the surface of the microcapsules, that is, those that were not encapsulated, were extracted according to the methodology described by Nori et al. (2011), with adaptations. Initially, 0.2 g of the microencapsulated powders were dissolved in 2.0 mL of pure ethanol. The mixture was then homogenized (30 s), centrifuged (4000× *g* for 2 min), and the supernatant was used for analysis. The encapsulation efficiency was calculated according to the equation below:

(3) $$\mathrm{EE}\;\left(\%\right)=\frac{\left(\mathrm{TP}-\mathrm{SP}\right)\times 100}{\mathrm{TP}}$$

where TP is the total phenolic compounds within microcapsules, and SP is the total phenolic compounds present on the surface.

### 2.7 Analysis of the Antioxidant Capacity of the Microcapsules

Antioxidant capacity analyses were performed on the microcapsules and on the extracts obtained to determine encapsulation efficiency. For these analyses, the microcapsules were solubilized in ultra‐pure water (MiliQ Direct 8) (0.1 g mL^−1^). The same extracts were analyzed for their antioxidant potential.

#### 2.7.1 Total Reducing Capacity (TRC)

TRC analysis was performed using the Folin–Ciocalteu method to estimate the content of total phenolic compounds. A 96‐well microplate reader (FlexStation III, Molecular Devices, CA, USA) was used for readings at 750 nm. The standard curve was constructed with gallic acid at known concentrations (5–130 mg L^−1^). The analysis was conducted in triplicate, and the results were expressed in mg of gallic acid equivalent (GAE) per 100 g of sample (Singleton et al. 1999).

#### 2.7.2 Scavenging Activity of the 1,1‐Diphenyl‐2‐Picrylhydrazyl Radical (DPPH)

DPPH radical scavenging activity was evaluated to measure the antioxidant potential of the samples according to Furlan et al. (2015), with modifications. Absorbances were recorded at 517 nm in a microplate reader (FlexStation III, Molecular Devices, California, USA). Sample (20 μL) and 280 μL of DPPH (80 μM) were mixed in a 96‐well plate (BMG Labtech 96, Germany) in triplicate and stored in the dark for 30 min. Antioxidant activity was calculated from linear regression of different concentrations of Trolox (6‐hydroxy 2,5,7,8‐tetramethylchroman‐2‐carboxylic acid) (0–10 μg mL^−1^). The radical scavenging activity was expressed as μg of Trolox equivalents (Eq.) per gram of microencapsulated powder and the percentage of antioxidant activity (%AAO).

$$\%\mathrm{AAO}=\frac{{\mathrm{Abs}}_{\text{DPPH}}-{\mathrm{Abs}}_{\text{sample}}}{{\mathrm{Abs}}_{\text{DPPH}}}\times 100$$

where: Abs_DPPH_ is the absorbance of the DPPH solution without extracts; Abs_sample_ is the absorbance of the sample solution.

#### 2.7.3 Determination of Antioxidant Activity by the Iron Reduction Method (FRAP)

The antioxidant activity assay by the FRAP method was conducted according to Furlan et al. (2015). The FRAP reagent solution was prepared with 50 mL of 0.3 M sodium acetate buffer (pH 3.6), 5 mL of 10 mM TPTZ (2,4,6‐tripyridyl‐s‐triazine), and 5 mL of 20 mM FeCl 3.6H_2_O. Subsequently, 20 μL of the sample was mixed with 15 μL of ultrapure water and 265 μL of the FRAP solution and incubated at 37°C for 30 min in the dark. Readings were performed at 593 nm using microplates. The Trolox standard curve at concentrations ranging from 0 to 7.5 μg mL^−1^ was used as a reference. The values obtained were expressed in μg TE per 100 g of sample.

#### 2.7.4 Oxygen Radical Absorption Capacity (ORAC)

ORAC analysis was determined using fluorescein in a microplate reader (Zulueta et al. 2009). As a control, 80 μL of the fluorescein solution (80 nM) and 80 μL of the samples were tested, and 75 mM sodium phosphate buffer (pH 7.4) was used as a blank. Next, 40 μL of AAPH (2,2′‐Azobis(2‐amidinopropane) dihydrochloride) (221 mM) was added. Analysis was monitored every 1 min for 90 min after the addition of AAPH in a microplate reader with a 485‐nm excitation filter and a 535‐nm emission filter. The antioxidant activity was expressed as an area under a time‐integrated fluorescence curve using GraphPad Prism software. The procedure was performed in triplicate. ORAC antioxidant activity was expressed as μg equivalents (Eq.) of Trolox per gram of microencapsulated infusion powder.

### 2.8 High‐Performance Liquid Chromatography (HPLC‐DAD)

The phenolic compound profile of the solubilized microcapsules (0.1 g mL^−1^) in ultrapure water (MilliQ Direct 8), was characterized by high‐performance liquid chromatography with a diode array detector (HPLC‐DAD), according to Gomes and Torres (2016), with adaptations. The samples were injected into the HPLC‐DAD system (Flexar, Perkin Elmer) using a reversed‐phase column (100‐5‐C18 with dimensions of 4.6 × 250 mm, Kromasil) operating at 40°C. The injected sample volume was 20 μL in triplicate at a flow rate of 0.8 mL/min, following a gradient of mobile phases A (ultrapure water containing 0.3% formic acid), B (100% methanol), and C (100% acetonitrile): 0.0 min—85% A; 14.5% B; 0.5% C; 7.0 min—55% A; 43.5% B; 1.5% C; 14.0 min—5% A; 93% B; 2% C; 20 min—1% A; 97% B; 2% C; 23 min—15% A; 83% B; 2% C; 23 to 33 min—85% A; 14.5% B; 0.5% C. The eluted components were detected at three different wavelengths: 260, 280, and 320 nm. The compounds were identified based on the retention times and absorption spectra obtained between 230 and 350 nm, comparing them with commercial reference standards. Table S1 presents the calibration curve of the phenolic compound standards used to identify the compounds in the samples, which presented precision parameters such as homoscedasticity and linearity deviation of *p* > 0.05 and significant regression of *p* < 0.001.

### 2.9 Statistical Analysis

The results were subjected to analysis of variance (ANOVA) with Tukey HSD post hoc test considering a significance level of 5% (*p* < 0.05). For this purpose, the XLSTAT statistical software (Addinsoft, 2019.1.1) was used.

## 3 Results and Discussion

Spray‐drying and freeze‐drying, as standard processes for obtaining powdered products, not only protect the compounds and increase the product's shelf life by eliminating much of the water but also open up exciting new possibilities for product application. These methods are efficient in the drying process, but it is important to understand their unique advantages and disadvantages, including the influence of temperature on morphology and physical–chemical and antioxidant properties. The cost of the process is a significant factor to consider, as it directly impacts the product's value. Spray‐drying, for instance, is a more cost‐effective option than freeze‐drying. However, it is worth noting that the high temperatures used in spray‐drying to form microcapsules can potentially affect some of their properties, as indicated by some studies (Ballesteros et al. 2017; Sun et al. 2020). Therefore, the two methods were chosen to evaluate the most advantageous drying method for the formulation of the pineapple crown soluble powder drink.

### 3.1 Physicochemical Characterization of Microcapsules

The moisture content, water activity, and hygroscopicity of microcapsules represent fundamental characteristics that influence the stability and storage efficiency of the encapsulated product. These aspects are related to viscosity, fluidity, drying efficiency, microbial growth, microstructure, and oxidation of bioactive compounds (Xiao et al. 2022). Table 1 presents the results of the physicochemical characteristics of the microencapsulated samples. The encapsulated, freeze‐dried (FD), and spray‐dried (SD) samples presented acidic and similar pH values (~pH 4.3, *p* < 0.05). The moisture contents were also similar (~3.5%, *p* < 0.05), within the ideal moisture range (below 5%) that ensures greater stability during storage for powdered products (Silva et al. 2023). The moisture content of microcapsules can directly impact their stability as the wall material transitions from the glassy form to the amorphous form at higher humidity levels, which can result in the release and degradation of the core material during storage (Kang et al. 2019).

**TABLE 1:** Physicochemical analyses of SD (spray‐dried) and FD (freeze‐dried) microcapsules.

	Moisture (%)	Aw	Solubility (%)	pH	Wettability (s)
SD	3.41 ± 1.14^a^	0.33 ± 0.01^a^	99.98 ± 0.003^a^	4.34 ± 0.05^a^	191 ± 22.52^a^
FD	3.59 ± 0.09^a^	0.26 ± 0.03^b^	99.98 ± 0.001^a^	4.38 ± 0.01^a^	1.0 ± 0.0^b^

*Note:* The results are expressed as mean ± standard deviation (*n* = 3). Different letters in the same column mean that there is a significant difference (*p* < 0.05) between the different samples.

The spray drying process achieves high temperatures and drying speeds, resulting in small particle sizes (≤ 100 μm) and low moisture content, generally allowing them to be stored for long periods (Silva et al. 2023). Microcapsules of cinnamon ( *Cinnamomum zeylanicum* ) infusion with maltodextrin obtained by spray drying revealed low moisture content (1.34%) (Santiago‐Adame et al. 2015). Cumac extract microcapsules with maltodextrin presented moisture content between 1.89% and 2.92% (Caliskan and Nur Dirim 2013). In the microencapsulation of green tea extract by spray drying using different proportions of maltodextrin with gum arabic and chitosan, moisture contents ranging from 2.3% to 4.8% were found, similar to those found in this study, especially for maltodextrin microcapsules (3.2%), indicating that the moisture contents of the microparticles may depend on the concentration and type of wall material used (Eun et al. 2020; Zokti et al. 2016).

It is noteworthy that decreasing moisture can increase its hygroscopicity since the absorption capacity is related to the moisture gradient between the powdered product and the surrounding atmosphere (Karrar et al. 2021). Although the moisture presented borderline values, the pH of the samples is sufficient to guarantee their conservation. In addition, the use of lower temperatures in the drying process tends to cause less damage to the final product. Moisture contents may vary due to the intrinsic characteristics of the samples, such as the presence of small pores that effectively resist mass transfer and act as a barrier against sublimation in freeze‐drying, their concentration, and wall material (Pasrija et al. 2015). The increase in the concentration of maltodextrin can cause a decrease in the moisture content of the microcapsules (Kang et al. 2019; Premi and Sharma 2017) compared to other wall materials. The higher degree of maltodextrin polymerization promotes better particles' drying and results in low moisture content in the final product. This can be explained by the presence of relatively few hydrophilic groups in this polymer that bind to fewer water molecules (Pasrija et al. 2015; Zokti et al. 2016). In addition, the low viscosity of maltodextrin positively impacts water diffusion during spray drying. On the other hand, the increase in moisture content in microencapsulated powders can also be caused by the high water retention capacity of the microcapsule core material (Xu et al. 2022). Yamashita et al. (2017) microencapsulated an anthocyanin‐rich blackberry (*Rubus* spp.) by‐product extract using maltodextrin with 10 and 20 dextrose equivalents (DE) as carriers through freeze‐drying, and observed that the moisture content decreased as the DE value increased. The water activity of the microcapsules was less than 0.40 (0.33 for SD and 0.26 for FD), indicating low susceptibility to microbial growth and degradation reactions, including hydrolysis, which may impair the quality and stability of the products (Silva et al. 2023). Green tea leaf extracts microencapsulated in a spray dryer obtained similar values, between 0.36 and 0.28 (Zokti et al. 2016). Caliskan and Nur Dirim (2013) obtained water activity values between 0.157 and 0.215 by microencapsulating sumac extract in maltodextrin by spray drying. The microcapsules were highly soluble (99.98%), presenting an excellent ability to dissolve in aqueous media, which is an important quality parameter for application in a product, as it facilitates its reconstitution and highlights the good result in the choice of wall material (Cao et al. 2020). Maltodextrin is the most widely used encapsulating agent due to its high solubility in water and low viscosity (Xiao et al. 2022). Similar results were found in mountain tea powders (*Sideritis stricta*) (98.5% to 99.5%) (Şahin Nadeem et al. 2011). In addition to the chosen wall material, the capsule core can interfere with solubility since hydrophobic compounds tend to have low solubility in water.

Wettability is one of the most important properties related to the reconstitution of powders in aqueous solutions and is directly affected by the molecular interaction between the two phases (Silva et al. 2018). In this study, the times obtained for the powders to become completely wet ranged from 1 to 191 s for FD and SD, respectively. Particle size was one of the factors that likely influenced the large difference in wettability between the two drying methods. Small particle sizes can lead to low wettability (Hogekamp and Schubert 2003). Very fine particles tend to form agglomerates (lumps). These agglomerates can resist wetting due to capillary forces or surface tension, leading to poor wettability. Besides, microencapsulation processes using spray‐drying with very high air inlet temperatures can form a rigid layer, which prevents the diffusion of water through the wall material, thus reducing wettability. On the other hand, the very light and highly porous irregular structure typical of freeze‐dried materials generates a large surface area, which can facilitate interaction with water, improving their wettability (Anwar and Kunz 2011). Caliskan and Nur Dirim (2013) obtained a much higher wettability value that ranged from 1239 s to 3263 s with microencapsulated sumac extracts in maltodextrin by spray drying. According to the authors, an increase in maltodextrin concentration in sumac extracts led to a significant decrease in wettability.

**FIGURE 1:**
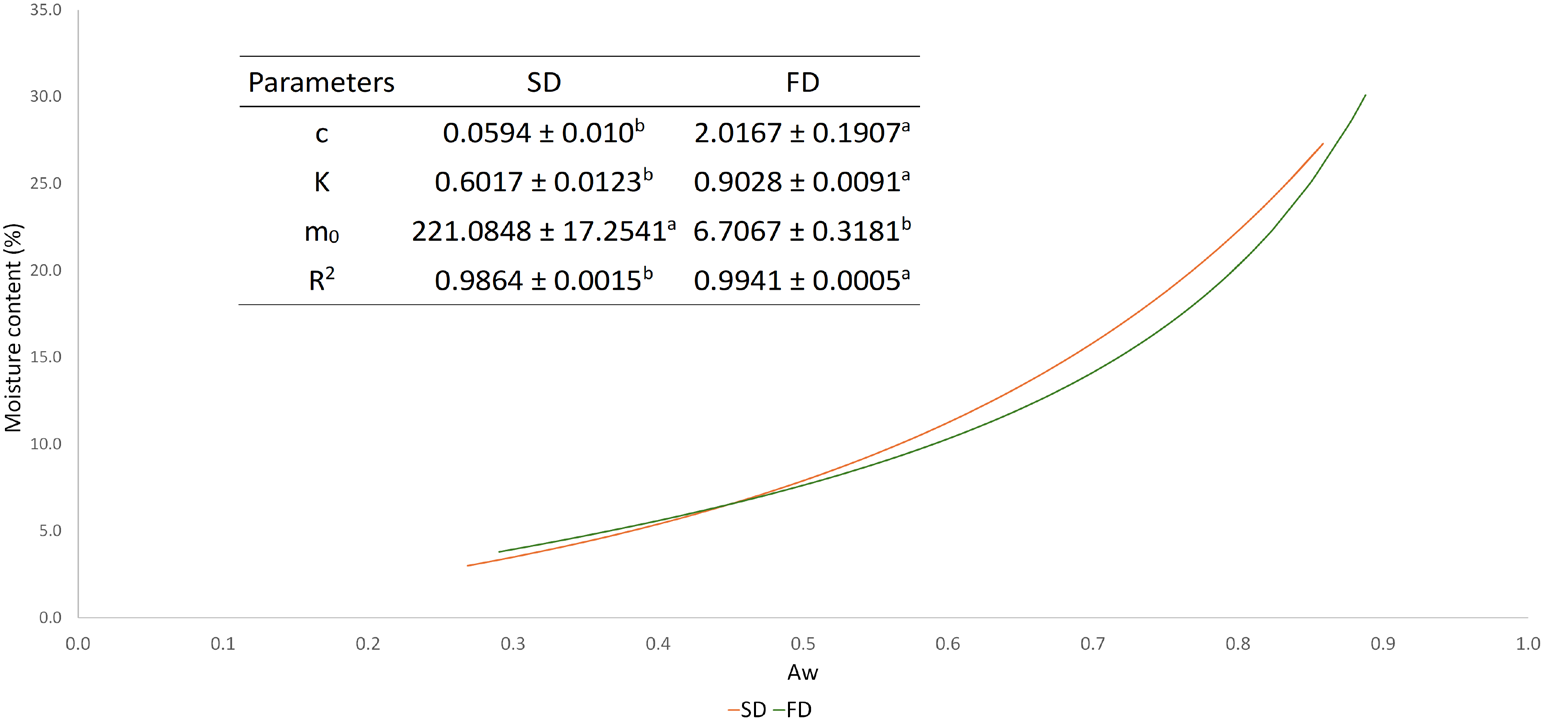
Curves and parameters of the sorption isotherms of the SD (spray‐drying) and FD (freeze‐dried) samples. Different letters in the same line represent a statistical difference (*p* < 0.01).

The construction of adsorption isotherms provides relevant information about the storage conditions and stability of the product obtained from the prediction of the interactions of water with this product (Edrisi Sormoli and Langrish 2016). Figure 1 shows the sorption isotherm curves of the two samples, revealing a J‐type curve characteristic of products with a composition rich in sugars and other solutes. It can be observed that from *a*_*w*_ 0.5, there is a difference in the slope of the curves, indicating that at the same humidity, the *a*_*w*_ of the FD sample is higher. It can be observed that the SD sample has a higher *m*_0_ value than the FD, indicating a more significant amount of water bound in the monolayer. The moisture content of the monolayer is an essential factor for correlating the stability of food because it indicates the amount of water directly bound to the active sites on the surface (Abrahão et al. 2019). This means that greater moisture can be adsorbed without altering the Aw of this sample. This suggests that this microencapsulated powder (SD) better supports moisture changes without losing storage stability. Maltodextrin contributes to the reduction of the surface exposed to water molecules, reducing the moisture content in the monolayer when compared to other wall materials (Hartwig et al. 2014). Regarding the *C* and *K* parameters, there were significant differences between the SD and FD samples, where the latter presented a higher *C* value (2.02), indicating that the water present is more strongly bound to the monolayer and there is a greater difference in enthalpy between the monolayer molecules and the subsequent layers (Arthur et al. 2018). For both types of drying, the modeling determination coefficients were *R* ^2^ > 0.98.

### 3.2 Particle Size and Diameter

The SD and FD samples showed significant differences in particle size, diameter, and distribution, as shown in Table 2. SD microcapsules presented significantly smaller sizes (*p* < 0.01) than FD, showing that the drying technique directly affected the particle size distribution. The D4.3 values for SD and FD were around 11.5 and 166.3 μm, respectively (Table 2). These values are in agreement with the literature for other powder materials using these drying techniques (Vardanega et al. 2019). Most (*d*_90_) of the particles produced by SD had a diameter of around 20 μm, while for the FD sample, the particles were 15 times larger. Therefore, it can be noted that the SD particles constitute a fine and clear powder, while the FD sample has larger granules consistent with the material that was particulate after the drying process. As reported by Cano‐Higuita et al. (2015), the variation in particle sizes is likely due to the specific processes associated with each drying technique. In spray drying, particle generation is influenced by the atomizer characteristics and the physical properties of the feed material, whereas in freeze drying, the ultimate particle size is determined by the grinding step. Chen et al. (2012) explained that the larger particle size of the freeze‐dried samples is caused by the low‐temperature process and the lack of resistance necessary to break the frozen droplets or alter the surface during drying. In addition, the particle size in SD drying, which is considerably smaller than in FD, may justify the different wettability times since tiny particles tend to form lumps, making them difficult to submerge. According to Yamashita et al. (2017) particle diameter of spray‐dried products ranges from 1 to 15 mm, whereas freeze‐dried products can reach 300 mm.

**TABLE 2:** Particle size distribution of microcapsules dried by spray‐drying (SD) and freeze‐drying (FD).

Parameters	SD	FD
D_4,3_ (μm)	11.79 ± 0.07^b^	166.29 ± 2.85^a^
d_10_ (μm)	2.22 ± 0.03^b^	43.54 ± 0.46^a^
d_50_ (μm)	10.85 ± 0.05^b^	135.65 ± 1.56^a^
d_90_ (μm)	21.73 ± 0.19^b^	331.61 ± 6.58^a^
Span (μm)	1.80 ± 0.02^b^	2.12 ± 0.03^a^

*Note:* Different letters in the same row represent significant difference with *p* < 0.05. D43: Average volume surface diameter; d10, d50 and d90 are the average volume diameters of 10%, 50% and 90% of the cumulative volume (μm) respectively; Span: particle polydispersity.

### 3.3 Morphological Characterization of Microcapsules

**FIGURE 2:**
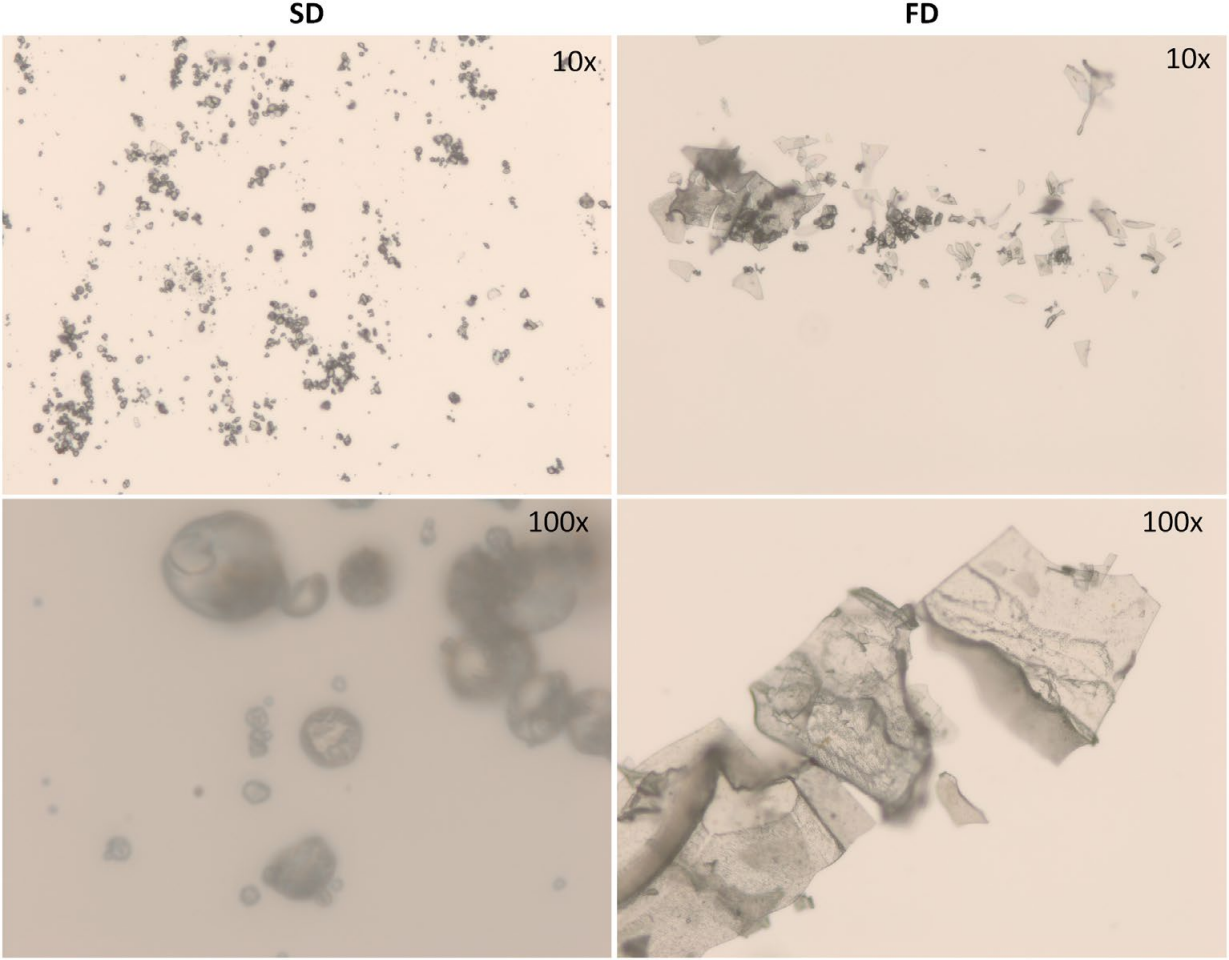
Fluorescence microscopy of microencapsulated pineapple crown flour infusions dried by spray‐drying (SD) and freeze‐drying (FD) viewed at different magnifications (10× and 100×).

Figure 2 shows the fluorescence micrograph of the microencapsulated infusions to observe the microparticles' appearance and distribution. It is possible to verify that the morphology of the FD sample is irregular, fragmented, and scaly, which is typical of the lyophilization process. In contrast, although presenting heterogeneous sizes, the SD sample has a predominantly spherical shape and smoother surfaces. These differences are due to the type of drying to which the samples were subjected. The surface of the FD particles is rougher and more heterogeneous, which is characteristic of the formation of pores during the sublimation process in lyophilization. Cano‐Higuita et al. (2015) observed that spray‐dried particles were predominantly spherical and uniform, with smooth, unbroken surfaces. These features are crucial for maintaining low gas permeability and ensuring better protection and retention of the encapsulated material. In contrast, freeze drying produced irregular, flake‐like particles with sharp, fractured, glassy surfaces, a characteristic attributed to the grinding process. This irregular, very light, and highly porous structure generates a large surface area, which can facilitate the diffusion of oxygen from the air on the surface of the particle, potentially decomposing compounds that are not protected or that are on the surface of the matrix (Anwar and Kunz 2011). Likewise, this may be why, in the wettability analysis, the FD sample took much less time than the SD sample for the powder to thoroughly wet.

**FIGURE 3:**
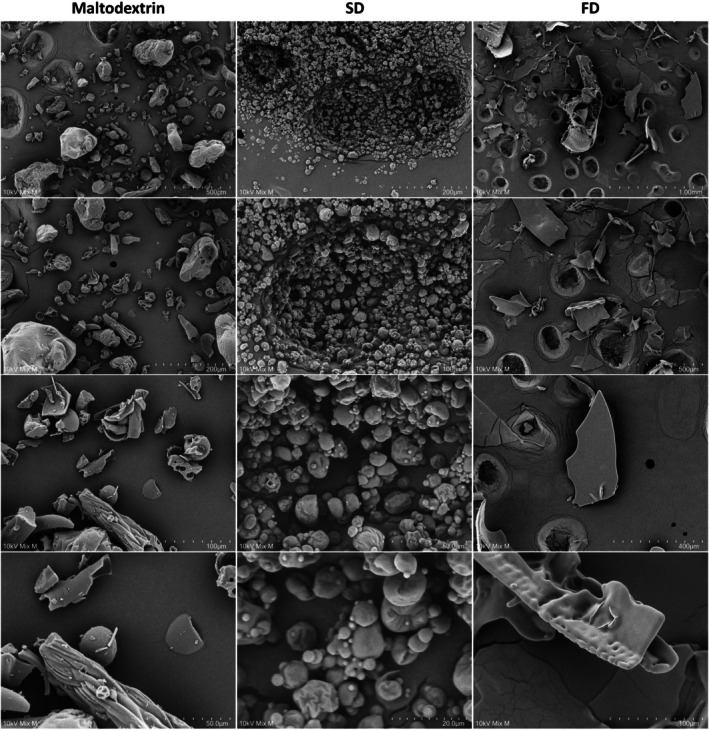
SEM images of maltodextrin and PCF infusion powder obtained by spray‐drying (SD) and freeze‐drying (FD).

The micrographs obtained by SEM (Figure 3) revealed large morphological differences in the SD and FD samples, showing that the drying process of the PCF infusion with maltodextrin can influence the microparticle structure. These results corroborate the analyses of particle size and distribution (Table 2) and fluorescence microscopy, where SD presented significantly smaller particles (15 times) than FD and with a heterogeneous distribution for both samples. Although greater solubility may be associated with a smaller particle size due to the greater surface area available for hydration (Kuck and Noreña 2016), both samples presented high levels of solubility due to their specific morphologies and composition of the wall and core material. It can be noted that the microparticles obtained by SD are spherical with a predominantly smooth surface and of varying sizes. As in the micrograph, the powder obtained by SD tends to form “lumps” that may be associated with its hygroscopicity. The microparticles obtained by FD have particulate characteristics, without a defined shape and with smooth surfaces, but in some parts, minor grooves typical of freeze‐drying can be seen (Chranioti et al. 2016). The effects of drying the PCF infusion corroborate the findings in ginger infusion powders obtained by spray‐drying, which presented irregular spherical particles, and the freeze‐dried ones irregular and flocculated particles (Vardanega et al. 2019).

### 3.4 Chemical Characterization by Fourier Transform Infrared Spectroscopy

**FIGURE 4:**
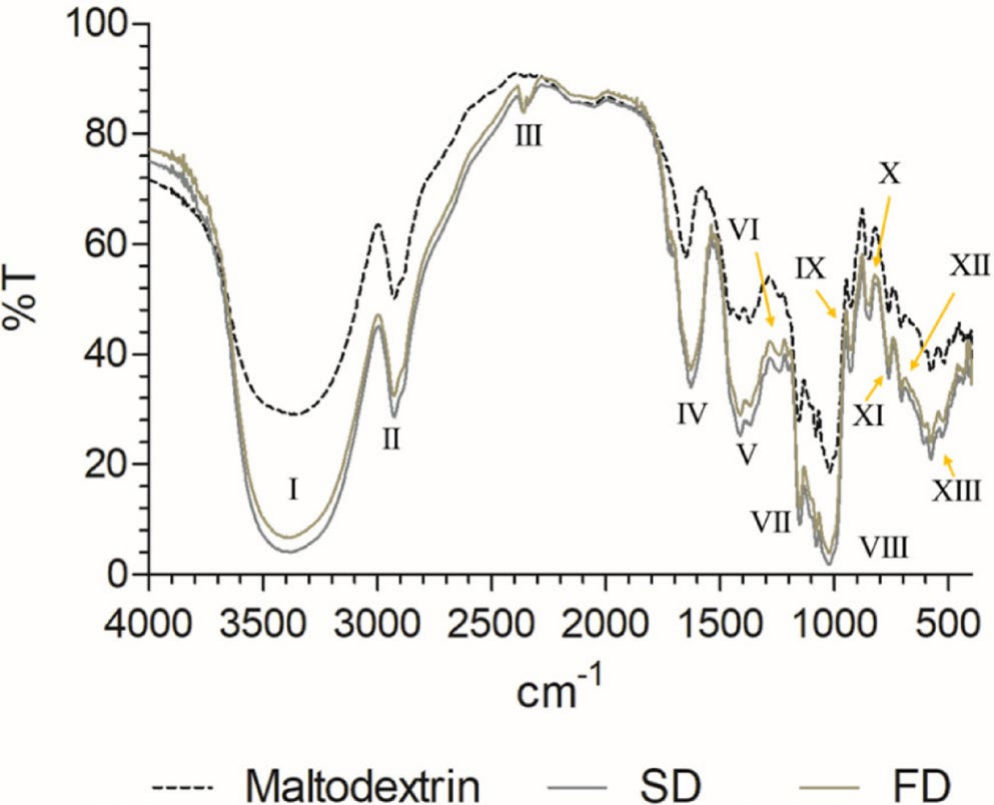
Fourier transform infrared spectroscopy spectra of maltodextrin, spray‐dried (SD) and freeze‐dried (FD) PCF infusion samples.

FTIR spectroscopy analysis was performed to better understand the chemical composition and functional grouping of the molecules present in the particles. The spectra of the SD and FD samples (Figure 4) were similar in their bands and transmittance (%T), revealing some differences in maltodextrin.

All spectra showed an absorption band at 3400 cm^−1^ (I), characteristic of hydroxyl groups (OH). However, it can be noted that the bands of the SD and FD samples increased to maltodextrin, indicating an increase in OH groups typical of polar bioactive compounds such as phenolic acids, for example. Characteristic peaks at 2900 cm^−1^ are attributed to the stretching vibration of the C‐H bond of alkanes. A small band was observed in the SD and FD samples (III) at 2350 cm^−1^ present in region 2 characteristic of C ≡ C bonds. This small band may indicate the presence of contaminants or impurities containing alkyne groups, degradation products of organic components during processing, or chemical interactions between the bioactive compounds of the pineapple crown infusion and the encapsulation material. In region 4, known as the fingerprint region of a substance, bands V–VIII indicate C‐O vibrations of functional groups such as carboxylic acid, alcohol, phenol, and ether (Table S2). In this same region, different wavelengths (600–900 cm^−1^) indicate the presence of aromatic rings (Nandiyanto et al. 2022). Overall, from the spectra evaluated by FTIR of the SD and FD samples, it can be suggested that the identified molecules agree with the composition of phenolic compounds present in the PCF used in the microencapsulated infusion (Brito et al. 2021).

### 3.5 Encapsulation Efficiency

High encapsulation efficiency was obtained for both samples, with 97% for the SD sample and 98.7% for the FD sample (Table S3). These values were higher than those found by Nori et al. (2011) in the encapsulation of propolis (72%), corroborating the results of the present study, since the total reducing compounds on the surfaces of both microcapsules were much lower (more than 30 times) than those found inside them. By determining the content of compounds after breaking the microcapsules, it is possible to conclude that the methodology used for both the SD and FD samples effectively retained the bioactive material from the pineapple crown flour infusion.

According to Akhavan Mahdavi et al. (2016), an encapsulation method can be considered adequate when it presents high retention of the core materials, ensuring that the amount of these materials present on the surface of the powder particles is minimal. This encapsulation efficiency is essential to ensure the stability and functionality of the encapsulated active compounds, contributing to their controlled release and protection against degradation. However, unlike the results of this study, these authors concluded that the exclusive use of maltodextrin as a wall material resulted in low encapsulation efficiency compared to samples that combined two different wall materials. The authors attributed the encapsulation efficiency between 86% and 93% to the lack of emulsification and the low film‐forming capacity of maltodextrin. Comparing microencapsulation techniques for phenolic extraction from carambola ( *Averrhoa carambola* ) bagasse by freeze drying and spray drying, a greater efficiency was found in freeze‐dried samples (78%–97%) compared to spray‐dried samples (63%–79%) (Saikia and Mahanta 2016), unlike the cited work, in the present study there was no significant difference.

### 3.6 Total Reducing Compound Content and Antioxidant Capacity

Several spectrophotometric assays were employed to evaluate the antioxidant activity of the samples solubilized in water (10%) and of the extracts used for encapsulation efficiency, since the analytical methods are based on different principles. Table 3 presents the results of the TRC and antioxidant capacity of the different samples microencapsulated with maltodextrin. It can be observed that the SD sample exhibited higher levels of total reducing capacity in relation to the FD sample. Similarly, the antioxidant activity of the SD sample did not present statistical differences in relation to the lyophilized sample in the FRAP and ORAC analyses.

**TABLE 3:** Antioxidant capacity and phenolic content of spray‐dried (SD) and freeze‐dried (FD) microencapsulated infusion.

Analysis	SD samples	FD samples
TRC (mg GAE. g^−1^)	10.16 ± 48.92^a^	8.84 ± 5.80^b^
FRAP (μg TE. g^−1^)	25.78 ± 3.33^a^	23.75 ± 3.38^a^
ORAC (μg TE. g^−1^)	77.74 ± 8.08^a^	62.34 ± 8.66^a^
*p*‐coumaric acid (μg. g^−1^)	55.87 ± 4.16^a^	23.81 ± 1.07^b^
*Trans‐*ferulic acid (μg. g^−1^)	11.40 ± 0.40^b^	15.08 ± 0.67^a^
Caffeic acid (μg. g^−1^)	23.0 ± 0.13^a^	n.d.

*Note:* The results are expressed as mean ± standard deviation (*n* = 3). Different letters show significant differences (*p* < 0.05) between the same line.

Abbreviations: FD: sample microencapsulated by freeze‐drying; n.d.: not determined; SD: sample microencapsulated by spray drying.

In the microencapsulation of pink pepper seed extract ( *Schinus terebinthifolius* ) incorporated into green propolis extract, lower TRC values were also found in the freeze‐dried microcapsules (18.25 mg GAE g^−1^) compared to the spray‐dried samples (25.15 mg GAE g^−1^). From these data, the authors concluded that the microcapsules produced by spray‐drying presented a greater amount of phenolic compounds released after reconstitution; that is, this technique better encapsulated and protected the bioactive compounds (Laureanti et al. 2023). The higher TRC values found in the SD sample compared to the FD can be explained by the need for a shorter processing time in spray drying, exposing the particles to drying conditions for a reduced period. Although higher inlet temperatures are used, the drying time is very short, so that the process occurs almost instantaneously (Laureanti et al. 2023; Rezende et al. 2018). In addition, the temperature inside the particle only reaches 40°C–70°C even with the high inlet temperature, as the water absorbs the latent heat of the particle during the phase change (liquid to gas), thus reducing the internal temperature. However, decomposition can occur independently (Nguyen et al. 2022). Similarly, microencapsulated samples of antioxidant phenolic compounds extracted from coffee grounds by freeze‐drying and spray‐drying using different coating materials obtained higher antioxidant activity by FRAP for the spray‐dried samples when compared to the freeze‐dried samples regardless of the wall material used. This can be explained by the sawdust‐like shape of the freeze‐dried powder samples with a higher surface area ratio than the microsphere shape of the spray‐dried sample. This results in greater exposure of active compounds in the freeze‐dried sample, which can lead to decomposition (Ballesteros et al. 2017).

As previously presented, the TRC determined by the Folin–Ciocateu method was higher after the rupture of the microparticles (corresponding to the interior) than on their surface. Consequently, the ruptured extracts of both samples presented greater antioxidant capacities by the two methods analyzed in comparison to the small value shown by the extractions of the compounds from the surface. These results reinforce what was found by EE%, which expresses the efficiency of retaining substances inside the capsules, demonstrating the deficient concentration of antioxidant compounds on the surfaces of the capsules, but 32 to 400 times higher inside them (after rupture).

### 3.7 Quantification of Phenolic Compounds by Liquid Chromatography (HPLC‐DAD)

PCF has an abundant composition of bioactive compounds, among which phenolic acids such as gallic acid, vanillic acid, transferulic acid, p‐coumaric acid, ferulic acid, caffeic acid, 4‐hydroxybenzoic acid and lignans stand out, as identified by liquid chromatography coupled to tandem mass spectrometry (UHPLC–MS/MS) analysis (Brito et al. 2021). Some of these compounds remained in the microencapsulated samples of the PCF infusion (Table 3).

Of the phenolic compound standards used for HPLC‐DAD analysis, it was possible to identify and quantify three in the microencapsulated samples and in the infusion, namely p‐coumaric, transferulic, and caffeic acids, belonging to the phenolic acid class and hydroxycinnamic acid subclass. The SD sample showed higher concentrations of p‐coumaric acid and caffeic acid compared to the other samples. The FD sample showed a higher level of transferulic acid; however, it was not possible to detect caffeic acid levels in the FD sample. This does not necessarily imply the absence of these compounds but may be attributed to the DAD detector's limitations or concentrations below the detection/quantification limit of the method used. The study by Steingass et al. (2015) found that the phenolic profile of pineapple crown includes isomeric esters of p‐coumaric acid or ferulic acid (p‐coumaroyl and ferulic aldarates), corroborating the phenolic acids found in the present study. The authors highlight the rarity in the literature of pepsides of hydroxycinnamic acids with hexaric acids found by the quantification of phenolic compounds by HPLC‐DAD, thus expanding the profile of identified phenolic compounds. In addition, the compound caffeoyl isocitrate, an ester of caffeic acid with isocitrate, was identified in the study.

Phenolic acids are widely used in the food, nutrition, pharmaceutical, and cosmetic industries (Badea and Radu 2018). Therefore, the presence of these acids is essential because they contribute to the sensory characteristics, functional properties, and quality of foods (Qiu et al. 2021). In the identification and quantification of phenolic acids and flavonoids in pineapple peel extracts by HPLC‐MS, peaks of gallic acid, catechin, epicatechin, and ferulic acid were observed, where the latter was found in values much higher than those of the present study (195 μg mL^−1^). However, it is worth mentioning that this analysis was performed with extracts from different parts of this fruit (Li et al. 2014).

p‐Coumaric acid has attracted considerable interest due to its diverse pharmacological and biological properties, such as cardioprotective, antioxidant, neuroprotective, antiulcer, antiplatelet, antimicrobial, chemoprotective, and anticancer activities (Pei et al. 2016). Studies have shown that this acid has significant potential as an anticancer agent, modulating inflammation, reducing oxidative stress, interrupting cell cycle progression, and inducing apoptosis in a series of cancer cell lines, such as colorectal cancer, presenting low toxicity to human health (Qiu et al. 2021; Roy et al. 2016). These properties make this acid a promising nutraceutical candidate for phytochemical‐based strategies to reduce the incidence and morbidity of colorectal cancer (Tehami et al. 2023). Furthermore, p‐coumaric acid has anti‐hyperglycemic activity mediated by delaying intestinal glucose absorption and increasing β‐cell activity, promoting greater insulin sensitivity (Abdel‐Moneim et al. 2022). Transferulic acid has antiproliferative and anti‐migratory effects in cancer cell lines and reduces intracellular reactive oxygen species and nitric oxide induced by hyperglycemia (Fong et al. 2016; Singh and Patil 2022). In addition, this acid also showed a potential protective effect against testicular damage induced by the well‐known chemotherapy drug cisplatin, widely used to treat various cancerous conditions. This suggests that transferulic acid can be considered a supplement in chemotherapy protocols (Hassanein et al. 2021). In addition, this acid showed significant anxiolytic activity, reducing locomotor activity in experimental animals and inducing calming behaviors (Bhuia et al. 2023). Caffeic acid is widely found in various natural sources, such as fruits, vegetables, tea, coffee, and oils. This compound and its derivatives have been used for centuries for their therapeutic and medicinal properties. It has a wide range of biological and pharmacological activities, including antioxidant, anti‐inflammatory, anticancer, and neuroprotective effects. Its potential therapeutic benefits are achieved mainly by suppressing and inhibiting transcription and cell growth factors (Alam et al. 2022; Mirzaei et al. 2021). Evidence reveals the ability of caffeic acid to reverse the effects of metabolic syndrome by reducing inflammatory markers such as TNFα and reducing oxidative stress parameters (Muhammad Abdul Kadar et al. 2021). As shown in Figure S1, other compounds are present in the samples, but it was impossible to identify them due to methodological limitations. Therefore, analyses with greater selectivity and sensitivity are indicated to evaluate these compounds better.

The study of food toxicity, mainly herbal and fruit infusions, is of great importance for consumer safety. Some pineapple crown toxicity tests have already been reported using different evaluation methods. The *Artemia salina* lethality test showed that the aqueous extract of pineapple crown flour (8%) did not present cytotoxic potential at any concentration evaluated (Brito et al. 2020a). This result corroborates the acute and subacute toxicity tests on aqueous extracts of pineapple crown using a rat model with doses ranging from 5 to 5000 mg/kg of body weight, where no signs of toxicity or mortality were observed (Dutta and Bhattacharyya 2013). The data obtained in this study on the composition of the microencapsulated pineapple crown infusion opens perspectives for future research, both in vivo and in vitro. The quantification of compounds such as p‐coumaric and transferulic acids can contribute to the existing literature by aiding the development of protocols that can maximize the antioxidant effect and other beneficial effects of these bioactive compounds in promoting human health.

## 4 Conclusion

Aiming at applying pineapple crown to obtain products and use as a food ingredient, microencapsulation was successfully obtained, with high encapsulation efficiency (~98%) of antioxidant compounds for both drying techniques. The spray drying technique provided spherical microcapsules with a predominantly smooth surface without fissures or cracks. On the other hand, the freeze‐dried samples were irregular, fragmented, and had a scaly appearance, typical characteristics of the process. Both materials obtained showed excellent solubility in water, with the freeze‐dried microcapsules showing greater wettability. The microcapsules obtained from pineapple crown infusion demonstrated excellent antioxidant activity and have a significant phenolic profile with the presence of hydroxycinnamic acids (i.e., p‐coumaric acid, trans‐ferulic acid, and caffeic acid) that have bioactive properties recognized in the literature.

Regarding the microencapsulation technique used, the sample obtained by spray drying showed better physicochemical, morphological, and antioxidant properties when compared to the lyophilized sample. Since spray‐drying is a lower‐cost process, it reinforces the advantages of this drying method for the formulation of powdered beverages, enabling a lower final cost product. This work is of great relevance, and the results can significantly enrich the existing literature. Due to their high antioxidant capacity, pineapple crown soluble powder drink can be considered high value‐added products. The powder produced has promising industrial characteristics and uses a part of the fruit that is discarded in conventional production practices. Therefore, this study highlights the functional and nutritional potential of pineapple crowns in formulating innovative products such as soluble powdered beverages from an agro‐industrial residue and suggests a sustainable solution for reducing waste in the food industry.

## Author Contributions

**J. L. Bicas:** conceptualization (equal), methodology (equal), resources (equal), supervision (equal), writing – review and editing (equal). **T. B. N. Brito:** conceptualization (equal), investigation (equal), methodology (equal), writing – original draft (equal). **M. S. L. Ferreira:** conceptualization (equal), funding acquisition (equal), methodology (equal), project administration (equal), resources (equal), supervision (equal), writing – review and editing (equal). **E. M. Antonio:** conceptualization (equal), investigation (equal), methodology (equal), writing – original draft (equal). **A. B. Koifman:** conceptualization (equal), investigation (equal), methodology (equal), writing – original draft (equal). **M. G. O. Carvalho:** conceptualization (equal), investigation (equal), methodology (equal). **F. S. N. Cardoso:** conceptualization (equal), methodology (equal), supervision (equal), writing – review and editing (equal). **A. E. C. Fai:** conceptualization (equal), funding acquisition (equal), investigation (equal), methodology (equal), project administration (equal), resources (equal), supervision (equal), writing – review and editing (equal).

## Conflicts of Interest

The authors declare no conflicts of interest.

## Supporting information


**Data S1:** fsn371220‐sup‐0001‐Supinfo.docx.

## Data Availability

Data sharing not applicable to this article as no datasets were generated or analyzed during the current study.
