# Supplementary material for: Turning Pineapple Crown Waste Into Value: Formulation and Analysis of an Instant Powder Beverage
Source: Food Sci Nutr. 2025 Nov 16;13(11):e71220. doi: 10.1002/fsn3.71220 (PMC12620562; doi:10.1002/fsn3.71220)
Supplement: Supplementary file 1 — Data S1: fsn371220‐sup‐0001‐Supinfo.docx. [file FSN3-13-e71220-s001.docx]

**Supplementary tables**

Supplementary table 1. Calibration curve of phenolic compounds analyzed by HPLC-DAD.

| **Phenolic compounds** | **Linear Range (µg. mL^-1^)** | **Equation** | **R²** |
| --- | --- | --- | --- |
| Caffeic acid | 1 a 15 | y = 140566x + 35203 | 0,9973 |
| 4-hydroxybenzoic acid | 1 a 20 | y = 151440x + 46215 | 0,9997 |
| Pyrogallol | 1 a 20 | y = 6486,8x + 2059,9 | 0,9993 |
| Chlorogenic acid | 1 a 30 | y = 73689x + 18324 | 0,9999 |
| p-Coumaric acid | 1 a 30  5 a 75 | y = 158768x + 5901  y =158768x + 5901 | 0,9999  0,9954 |
| Transferulic acid | 1 a 75 | y = 135844x + 7055,5 | 0,9995 |

Supplementary table 2. Groups identified in the spectra obtained by Fourier Transform Infrared Spectroscopy (FTIR) of the samples.

| **Band** | **Frequency (cm^-1^)** | **Type of vibration** |
| --- | --- | --- |
| I | 3400-3200 | O-H |
| II | 2960-2850 | C-H (aliphatic) |
| III | 2350-2300 | (C≡C) |
| IV | 1620-1600 | C=C (aromatic) |
| V | 1420-1200 | C-O (carboxylic acid) |
| VI | 1230-1250 | C-O (carboxylic acid) |
| VII | 1200-1050 | C-O (alcohol and phenols) |
| VIII | 1020 | C-O; C-O-C |
| IX | 900 | Aromatic ring |
| X | 850 | Aromatic ring |
| XI | 750 | Aromatic ring |
| XII | 700 | Aromatic ring |
| XIII | 600 | Aromatic ring |

Supplementary table 3. Encapsulation efficiency (EE%), total reducing compound content and antioxidant capacity (DPPH and FRAP) of microcapsules dried by spray-dying and freeze-drying after disruption and extraction of phenolic compounds from the surface

| **Samples** | **EE%** | **TRC (****mg GAE. 100 g^-1^)** | **DPPH (µg TE. g^-1^)** | **FRAP (µg TE. g^-1^)** |
| --- | --- | --- | --- | --- |
| **RS** | 96.99% | 9397.27 ± 562.67^a^ | 416.52 ± 12.24^a^ | 161.31 ± 19.84^a^ |
| **SS** |  | 282.47 ± 48.50^b^ | 13.48 ± 6.47^b^ | 4.36 ± 1.21^b^ |
| **RF** | 98.66% | 8908.73 ± 367.39^a^ | 420.64 ± 8.98^a^ | 150.15 ± 24.16^a^ |
| **SF** |  | 127.35 ± 4.33^b^ | 0.37 ± 0.00^b^ | 8.45 ± 3.38^b^ |
| The results are expressed as mean ± standard deviation (n=3). Different letters in the TRC (total reducing capacity), DPPH and FRAP columns indicate significant difference (p<0.05) between the means. RS: extract of ruptured spray-drying microcapsule; SS: extract of compounds adhered to the surface of spray-drying microcapsules. RF: extract of ruptured freeze-dried microcapsule; SF: extract of compounds adhered to the surface of freeze-dried microcapsule. | | | | |

**Supplementary figure**


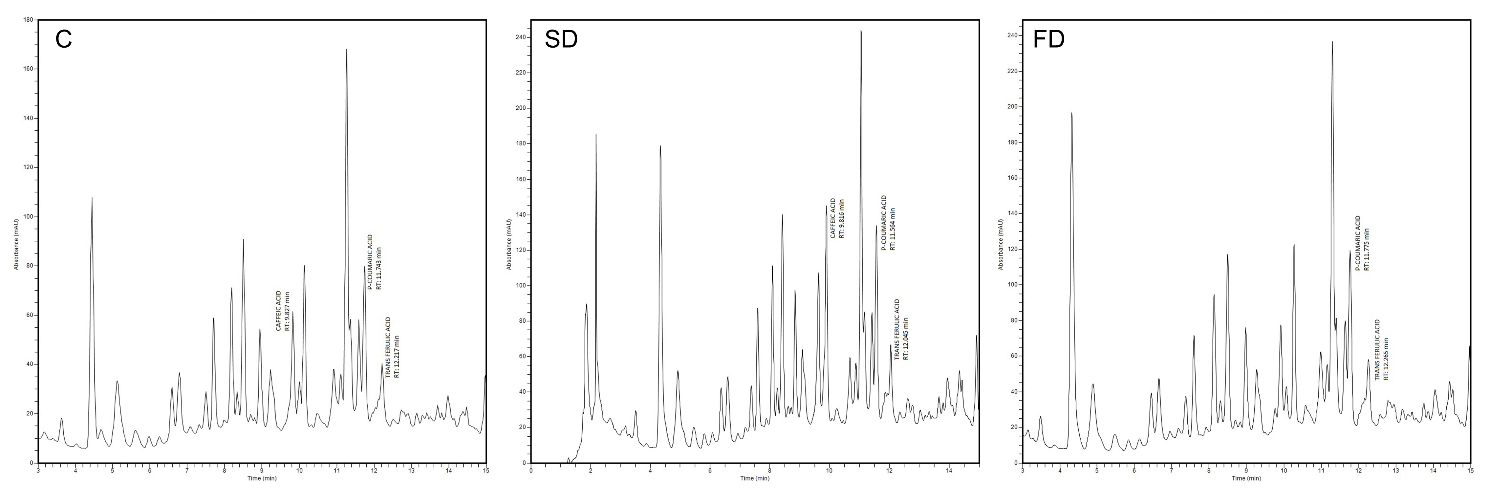


Supplementary figure 1. Chromatograms of the infusion samples of liquid PCF (C), microencapsulated by Spray-drying (SD) and Lyophilization (FD).
